# Synthetic Source Universal Domain Adaptation through Contrastive Learning

**DOI:** 10.3390/s21227539

**Published:** 2021-11-12

**Authors:** Jungchan Cho

**Affiliations:** School of Computing, Gachon University, Seongnam 13120, Korea; thinkai@gachon.ac.kr; Tel.: +82-31-750-5328

**Keywords:** universal domain adaptation, contrastive learning, classification, deep learning

## Abstract

Universal domain adaptation (UDA) is a crucial research topic for efficient deep learning model training using data from various imaging sensors. However, its development is affected by unlabeled target data. Moreover, the nonexistence of prior knowledge of the source and target domain makes it more challenging for UDA to train models. I hypothesize that the degradation of trained models in the target domain is caused by the lack of direct training loss to improve the discriminative power of the target domain data. As a result, the target data adapted to the source representations is biased toward the source domain. I found that the degradation was more pronounced when I used synthetic data for the source domain and real data for the target domain. In this paper, I propose a UDA method with target domain contrastive learning. The proposed method enables models to leverage synthetic data for the source domain and train the discriminativeness of target features in an unsupervised manner. In addition, the target domain feature extraction network is shared with the source domain classification task, preventing unnecessary computational growth. Extensive experimental results on VisDa-2017 and MNIST to SVHN demonstrated that the proposed method significantly outperforms the baseline by 2.7% and 5.1%, respectively.

## 1. Introduction

The application of deep learning has been rapidly improving performance in various fields of computer vision, such as object detection [1], human pose estimation [2,3], semantic segmentation [4,5], and image classification [6]. In addition, its application in real-world industries has also been promoted [7,8].

Achieving significant improvement in deep learning relies on the abundance of labeled samples [9] in a supervised manner. However, since images are collected from various sensors, test samples may be different from those used in the training phase. In this case, even if the test sample is semantically the same as the training samples, the performance is significantly reduced [10]. This is known as the domain shift problem [11]. As a result, deep learning models require new training for a new domain. Training data must be collected again, and the labeling process must be repeated continuously even for the same task in different domains. By contrast, humans are capable of robustly recognizing images and inferring their meanings across different domains. For example, a person who has seen and understood pictures of numerous cars (source domain) can also recognize them on artwork depicting cars (target domain). Hence, recent studies on deep learning have focused on increasing the efficiency of training models by leveraging information that has already been learned [12].

Domain adaptation (DA) [13,14] is the task of adapting algorithms trained on one or more source domains to other related target domains in the same task. Many studies transferred the knowledge learned from the source domain to the target domain successfully without target data labels. However, there is a common assumption that the label sets of the source and target domains are the same, i.e., closed-set domain adaptation (CDA) [15,16,17,18]. However, closed-set domain adaptation cannot effectively bridge label gaps in different domains if the classes in the target domain are less than those in the source domain, i.e., partial domain adaptation (PDA) [19,20,21], or if the target domain includes “unknown” classes that are not visible in the source domain, i.e., open-set domain adaptation (ODA) [22,23,24].

Currently, research is underway on how to adapt learned knowledge to different domains with no restrictions to the source and target domain label sets. Universal domain adaptation (UDA) [25,26] includes CDA, PDA, and ODA. As a result, target domain samples should be correctly classified into “known” class labels in a source domain or “unknown” classes if the label is not in the source domain [25,26]. Saito et al. [26] achieved UDA by proposing neighbor clustering and entropy separation in a self-supervised manner. Neighbor clustering brings target samples closer to known source class prototypes or unknown target samples. At the same time, entropy separation separates unknown target samples from known class boundaries using confident target samples.

**Figure 1:**
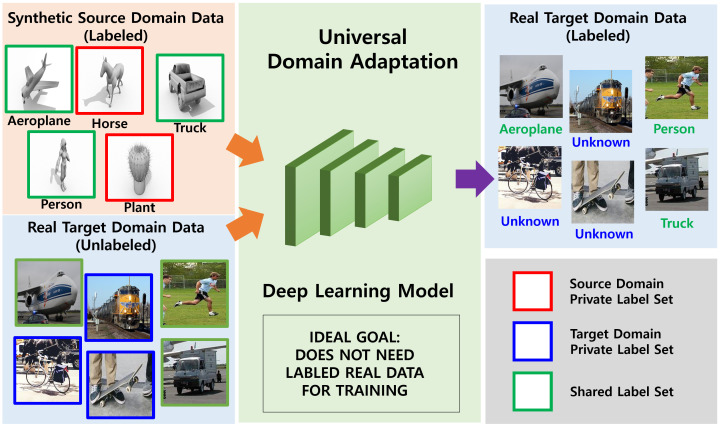
In ideal circumstances, universal domain adaptation should apply the knowledge learned from synthesized source domain data to the real target domain. Therefore, I focus on leveraging synthetic source domain data to reduce labeling costs by humans.

Even though [26] demonstrated a compelling performance for universal domain adaptation, I found that it is insufficient when synthesized data were used as a source domain. My hypothesis on this problem is that if the source and target domains are too different, self-supervision by two losses is erroneous, and the feature extraction network trained by [26] does not obtain discriminative feature representations for the target domains. This drawback stems from the fact that there is no direct training loss for the target domain. Ideally, universal domain adaptation should be able to adapt knowledge learned from synthesized source domain data to the real-world target domain, as depicted in Figure 1. It is a fundamental solution that can automatically generate labeled training data without human intervention, thereby reducing the cost of large-scale data labeling.

In this paper, I propose a novel universal domain adaptation method that improves the discriminative power of the target feature representation of [26]. Here, discriminative means that positive samples are pulled together, and multiple negative samples are pushed toward each other. However, because there are no labels available in the target domain, the proposed method utilizes contrastive learning [27] for target domain training, wherein I first generate a positive pair by augmenting the same target domain sample. Then, negative samples are generated by randomly selecting samples from the target minibatch except for the positive pair. By following this process, I can maximize the mutual information between different views on the same data in the target domain without requiring any labels for positive pairs. Moreover, the improved discriminative power in the target domain helps neighborhood clustering and known/unknown separation. Thereby, the proposed method achieves better performance than the baseline method [26] when a synthetic source domain is adapted to a real target domain.

The contributions are summarized as follows:

- In contrast to the existing universal domain adaptation methods [25,26], I focused on using synthetic source domain data to reduce human labeling costs.
- In this case, owing to the limitations of data synthesis, the source domain has different characteristics from the real data in the target domain. Thus, the target domain information should be fully utilized to learn the feature representation. I used contrastive learning [27] to extract discriminative features from the target domain.
- For contrastive learning, the target domain feature extraction network is not separately constructed. The source and target domains share a common feature extraction network, thus avoiding unnecessary computation surges.
- The experiments conducted on the VisDa-2017 dataset and MNIST to SVHN dataset indicate that the proposed method significantly outperforms baselines by 2.7% and 5.1%, respectively.

The remainder of this paper is organized as follows: Section 2 introduces related work. In Section 3, the proposed method is described. Section 4 presents the implementation and experimental results. Finally, Section 5 concludes the paper.

## 2. Related Work

### 2.1. Domain Adaptation

Let $L_{s}$ and $L_{t}$ denote a set of class labels present in the source and target, respectively. Considering this, domain adaptions can be categorized into three main topics according to the label set constraints between domains:

- Closed-set domain adaptation, if $L_{s}=L_{t}$. The main challenge in closed-set domain adaptation is to mitigate the domain gap between the source and target domains.
- Partial domain adaptation, if $L_{s}⊃L_{t}$. This does not assume that label sets are identical across domains, but that the source label set subsumes the target label set. The mismatched label set in the source domain presents a new challenge for domain adaptation.
- Open-set domain adaptation, if $L_{s}\subset L_{t}$. To avoid the unrealistic assumptions of closed-set domain adaptation, open-set domain adaptation assumes that the target domain contains unknown labels in the source domain.

I here introduce recent domain adaptation methods.

#### 2.1.1. Close-Set Domain Adaptation

Haeusser et al. [28] produced similar feature representations of the source and target domains by utilizing a bipartite graph. Tzeng et al. [29] first outlined a unified framework for adversarial domain adaptation by combining discriminative modeling, untied weight sharing, and generative adversarial loss. Long et al. [30] designed a conditional domain adversarial network with multilinear and entropy conditioning to improve discriminability and transferability.

#### 2.1.2. Partial Domain Adaptation

Cao et al. [19] introduced partial domain adaptation as a new challenge and proposed a down-weighting solution to handle outlier source classes that do not appear in the target domain. Cao et al. [20] further improved PDA and proposed an example transfer network that was designed using a weighting scheme to quantify the transferability of examples in the source domain. Therefore, it alleviates negative transfers and promotes positive transfers. Zhang et al. [21] extended the adversarial nets-based domain adaptation that identifies the importance score of source samples based on a two-domain classifier strategy.

#### 2.1.3. Open-Set Domain Adaptation

Busto et al. [22] introduced the concept of open sets to domain adaptation and proposed a method to fit in both closed and open-set scenarios by solving the assignment problem of targets that are potentially known classes in the source domain. Saito et al. [23] proposed a method for learning feature representations that separate unknown targets from known target samples based on adversarial training.

Recently, You et al. [25] introduced a universal domain adaptation and proposed a universal adaptation network that enables the shared label to be distinguished from the private label set for each domain by quantifying sample-level transferability. Satio et al. [26] improved UDA by proposing neighboring cluster and entropy separation losses, which are trained in a self-supervised manner. However, I found that this method [26] was insufficient when synthesized samples were used for the source domain. In this paper, I propose a contrastive-based UDA method for leveraging synthetic source data.

### 2.2. Contrastive Learning

Recent studies have shown that unsupervised feature representations for various downstream tasks can be learned in a contrastive manner. Oord et al. [31] captured useful feature representation by predicting the future in latent space based on autoregressive models and probabilistic contrastive loss. MoCo [32] is a seminal method for contrastive learning that maintains a dynamic dictionary (memory bank) for computing the contrastive loss and shows competitive results on ImageNet [9] classification. Chen et al. [27] conducted systematic studies to understand the factors that enable contrastive prediction tasks to learn useful representations. Grill et al. [33] achieved an improved accuracy of the ImageNet classification task without negative pairs. Instead, they relied on online target networks that interact and learn from each other.

Based on the abovementioned success of contrastive learning, I extracted the target domain information in a contrastive manner for universal domain adaptation.

## 3. Approach

### 3.1. Notation

Let the dataset for the source domain be $D^{s}={\left\{x_{i}^{s},y_{i}^{s}\right\}}_{i=1}^{N^{s}}$, where $x_{i}^{s}$ and $y_{i}^{s}$ indicate the *i*-th input sample and its corresponding true label, respectively. $N^{s}$ is the number of samples in the source domain. The target domain dataset $D^{t}={\left\{x_{i}^{t}\right\}}_{i=1}^{N^{t}}$ does not have true labels in which samples $x^{t}$ correspond to the same classes or unknown samples in the source domain. Let ${\tilde{x}}_{i}^{s}$ indicate augmented source domain samples by data augmentation μ, e.g., random cropping, random color distortion, etc. The feature representation ${\tilde{f}}_{i}^{s}\in R^{d}$ is calculated using a feature extraction network *G*, i.e., ${\tilde{f}}_{i}^{s}=G\left({\tilde{x}}_{i}^{s}\right)$. For the target domain, two different data augmentations μ and $\mu^{\prime }$ are applied to the target domain sample $x_{i}^{t}$. The augmented samples are denoted by ${\tilde{x}}_{i}^{t}$ and ${\hat{x}}_{i}^{t}$, and their feature representations by *G* are denoted by ${\tilde{f}}_{i}^{t}$ and ${\hat{f}}_{i}^{t}$. The same feature extraction network *G* is shared for both the source and target domains. Let C(·;W) be a linear classification network, where the weights are represented as $W=[w_{1},w_{2},\ldots ,w_{K}]$. The *k*-th weight vector $w_{k}\in R^{d}$ is normalized by the $l_{2}$ norm and used as a prototype representing the *k*-th class. The proposed method uses a memory bank ${\tilde{F}}^{t}=\left[{\tilde{f}}_{1}^{t},{\tilde{f}}_{2}^{t},\ldots ,{\tilde{f}}_{N^{t}}^{t}\right]\in R^{d\times N^{t}}$ that saves $N^{t}$ feature representations of samples in the target domain. I also denote the total memory bank as $V=\left[{\tilde{F}}^{t},W\right]=\left[{\tilde{f}}_{1}^{t},{\tilde{f}}_{2}^{t},\ldots ,{\tilde{f}}_{N^{t}}^{t},w_{1},w_{2},\ldots ,w_{K}\right]\in R^{d\times (N^{t}+K)}$.

### 3.2. Architecture

In this study, domain adaptation aims to transfer the knowledge of labeled samples in the source domain to train a classification model for unlabeled target domain samples. However, the problem is made more challenging for universal domain adaptation by including unknown samples in the target domain. Furthermore, some classes in the source domain may not have samples in the target domain. Therefore, the feature extraction network should move target domain samples closer to known class samples from the source domain, simultaneously making it easier to distinguish between unknown and known samples. To achieve this goal, researchers in a previous work (DANCE) [26] trained the feature extraction network through two losses: neighbor clustering and entropy separation. However, there is no direct loss function available to learn a target-domain-specific representation due to a lack of true labels for the target domain sample. My hypothesis is that the absence of the target domain-specific loss function causes the feature extraction network to create biased feature representations for source domain classification. This prevents useful feature representation for unknown target domain samples. To resolve this, I propose a method that learns feature representation for a source domain classification task in a supervised manner and a target domain instance-level classification task in an unsupervised manner.

**Figure 2:**
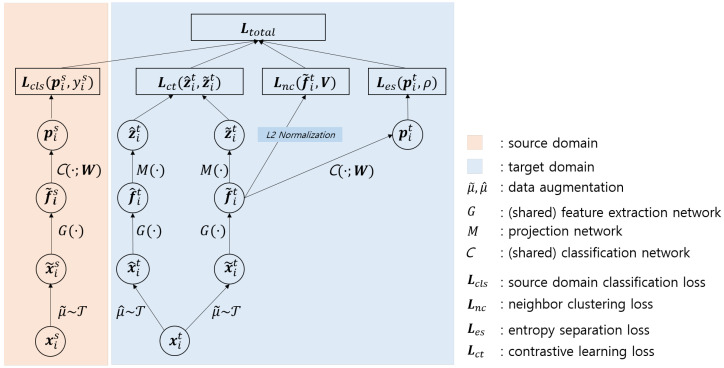
Overview. The proposed method learns a feature representation space considering the discriminative power in both the source ($L_{cls}$) and target ($L_{ct}$ in Section 3.2.1) domains. The target domain feature is enhanced using contrastive learning, which is an instance-level classification task to overcome the limitations of data synthesis in the source domain and unlabeled target domain data. The remaining losses ($L_{nc}$ in Section 3.2.2 and $L_{es}$ in Section 3.2.3) cluster class samples and separate known/unknown classes, respectively. Notably, the feature extraction network *G* is shared in the source and target domains. T denotes the pool of data augmentation.

To summarize, the proposed method consists of four loss functions as depicted in Figure 2. The first function is the loss function for source domain classification ($L_{cls}$), the second function is the instance-level classification of the target domain ($L_{ct}$), and the other two functions are the losses for neighbor clustering ($L_{nc}$) and entropy separation ($L_{es}$) proposed in [26]. I sequentially explain three loss functions, except for the source domain classification loss function.

#### 3.2.1. Target Domain Contrastive Loss

Contrastive representation learning aims to learn a discriminative embedding space devoid of any true labels through self-supervised learning wherein similar pairs of samples are close to each other, and dissimilar pairs of samples are distant from each other. To achieve this, each image in a given dataset is considered its own class, i.e., instance-level classification [27].

I used the SimCLR-based contrastive learning method [27] to learn the target domain feature representation. The procedure followed is as given below.

- Generate different perspectives based on the same target sample:Randomly selected data augmentations μ and $\mu^{\prime }$ are applied sequentially to a target domain sample $x_{i}^{t}$, obtaining two augmented samples ${\tilde{x}}_{i}^{t}$ and ${\hat{x}}_{i}^{t}$. As a result, two sets of minibatches are obtained: ${\tilde{B}}^{t}=\left\{\tilde{x_{k}^{t}}\right\}$ and ${\hat{B}}^{t}=\left\{\hat{x_{k}^{t}}\right\}$. The size of each minibatch is equal, i.e., $|{\tilde{B}}^{t}\left|=\right|{\hat{B}}^{t}|$.
- Extract features from augmented target samples: The augmented target samples are fed into a feature extraction network *G* to generate feature representations ${\tilde{f}}_{i}^{t}$ and ${\hat{f}}_{i}^{t}$. The feature extraction network is shared for the source-domain classification task. Although any network is freely available, I use ResNet [6,27], i.e.,
  (1) $$\begin{array}{cc} {\tilde{f}}_{i}^{t} & =G\left({\tilde{x}}_{i}^{t}\right)=ResNet\left({\tilde{x}}_{i}^{t}\right),\\ {\hat{f}}_{i}^{t} & =G\left({\hat{x}}_{i}^{t}\right)=ResNet\left({\hat{x}}_{i}^{t}\right). \end{array}$$
- Obtain target projection features for contrastive learning: Projection feature representations ${\tilde{z}}_{i}^{t}$ and ${\hat{z}}_{i}^{t}$ are obtained via a projection network *M*, and contrastive learning loss is applied to them. I use a shallow multilayer perceptron (MLP) for projection networks *M*, i.e.,
  (2) $$\begin{array}{cc} {\tilde{z}}_{i}^{t} & =M\left({\tilde{f}}_{i}^{t}\right)=MLP\left({\tilde{f}}_{i}^{t}\right),\\ {\hat{z}}_{i}^{t} & =M\left({\hat{f}}_{i}^{t}\right)=MLP\left({\hat{f}}_{i}^{t}\right). \end{array}$$
- Minimize contrastive learning loss in the target domain: Given two augmented minibatches ${\tilde{B}}^{t}$ and ${\hat{B}}^{t}$, contrastive learning aims to identify ${\hat{x}}_{i}^{t}$ using ${\tilde{x}}^{t}$ or vice versa. The loss function is defined using the projection representation of the target samples as follows:
  (3) $$\begin{array}{cc} L_{ct} & =\frac{1}{2|{\tilde{B}}^{t}|}\Sigma_{i=1}^{|\tilde{B}|}\left[L_{ict}\left({\tilde{z}}_{i}^{t},{\hat{z}}_{i}^{t}\right)+L_{ict}\left({\hat{z}}_{i}^{t},{\tilde{z}}_{i}^{t}\right)\right],\\ \; & L_{ict}\left({\tilde{z}}_{i}^{t},{\hat{z}}_{i}^{t}\right)=-\log\frac{\exp(s\left({\tilde{z}}_{i}^{t},{\hat{z}}_{i}^{t}\right)/\tau )}{Z}, \end{array}$$
  where τ is the temperature parameter [34].
  $Z=\Sigma_{k=1}^{N^{t}}\exp\left(s\left({\tilde{z}}_{i}^{t},{\hat{z}}_{k}^{t}\right)/\tau \right)+\Sigma_{k=1,k\neq i}^{N^{t}}\exp\left(s\left({\tilde{z}}_{i}^{t},{\tilde{z}}_{k}^{t}\right)/\tau \right)$, and $s({\tilde{z}}_{i}^{t},{\hat{z}}_{i}^{t})$ is the similarity function. I used the cosine similarity for this as $s\left(\tilde{z_{i}^{t}},{\hat{z}}_{i}^{t}\right)=\frac{{\tilde{z_{i}^{t}}}^{T}{\hat{z}}_{i}^{t}}{\left|\right|{\tilde{z}}_{i}^{t}\left|||\right|{\hat{z}}_{i}^{t}\left|\right|}$.

Minimizing the contrastive learning loss function causes the two projection feature representations from the same sample to be similar and the feature representations from different samples to be dissimilar. This results in learning powerful feature representations for instance-level classification, even without any labeled samples. Thus, the contrastive learning loss finds a feature space that represents better target domain samples by eliminating unnecessary information.

#### 3.2.2. Neighbor Clustering (NC) Loss

The purpose of this loss function [26] is to bring target samples together in the unknown class prototypes in the source domain or locate them close to their neighborhood in unknown target domain samples. This can be achieved by minimizing the following entropy function:

(4) $$L_{nc}=-\frac{1}{|{\tilde{B}}^{t}|}\Sigma_{i=1}^{|{\tilde{B}}^{t}|}\;\Sigma_{j=1,j\neq i}^{N^{t}+K}\;p_{i,j}\log\left(p_{i,j}\right),$$

where $p_{i,j}$ is a probability based on the similarity between the *i*-th target feature representation ${\tilde{f}}_{i}^{t}$ and the *j*-th total memory bank element $v_{j}$ as

(5) $$p_{i,j}=\frac{\exp(v_{j}^{T}{\tilde{f}}_{i}^{t}/\tau )}{\Sigma_{j=1,j\neq i}^{N^{t}+K}\exp\left(v_{j}^{T}{\tilde{f}}_{i}^{t}/\tau \right)},$$

where τ is the temperature parameter to control the distribution concentration degree [34].

#### 3.2.3. Entropy Separation Loss

Even with neighbor clustering loss, it is challenging to separate unknown samples from known classes. To enhance separation, the entropy separation loss function proposed in [26] is minimized.

(6) $$\begin{array}{cc} \; & L_{es}=\frac{1}{|{\tilde{B}}^{t}|}\Sigma_{i=1}^{|\tilde{B}|}L_{ies}\left(p_{i}\right),\\ L_{ies} & =\left\{\begin{array}{cc} -|H(p_{i})-\rho | & (|H\left(p_{i}\right)-\rho |>m),\\ 0 & otherwise \end{array}\right. \end{array}$$

where $p_{i}$ is a source class probability vector for the *i*-th target sample, which is calculated as $p_{i}=W^{T}{\tilde{f}}_{i}^{t}$. H(·) is the entropy function. If the value $H(p_{i})$ is much lower, it becomes nearly indistinguishable to one particular class in the source domain (low entropy), whereas if it is much higher, there are no similar classes in the source class (high entropy). Thus, the extremes entropy value $H(p_{i})$ is obtained by minimizing the entropy separation loss. This creates a separation effect in which the unknown samples move away from the class of the source domain. ρ is a threshold boundary value used to separate unknown samples from known classes, and *m* is a tuning parameter for minimizing the loss function using only reliable samples.

#### 3.2.4. Total Loss

Finally, I combine all four loss functions described above with two hyperparameters, $\lambda_{1}$ and $\lambda_{2}$, as follows:

(7) $$L_{total}=L_{cls}+\lambda_{1}L_{ct}+\lambda_{2}\left(L_{nc}+L_{es}\right).$$

Minimizing the total loss function results in learning the discriminative feature representation for both source and target domains, neighborhood clustering in the feature space, and maximizing the separation of unknown samples from known classes.

## 4. Experiments

### 4.1. Implementation Details

I conducted experiments in PyTorch [35] and on a single NVIDIA Titan RTX GPU and followed experimental settings [26]. However, the proposed method is not hardware dependent. The feature extractor *G* was set to ResNet50 [6] and pretrained on ImageNet [9] after removing the last linear layer in all experiments. In addition, I added a new source classification layer W. For contrastive projection features, I used a two-layer perception with ReLU activation, i.e., Linear-ReLU-Linear. For baselines, I used the implementation of a previous work [26]. For the proposed method, the values of ρ, *m*, and $\lambda_{1}$ were set to log(K)/2, 0.5, and 0.05, respectively, where *K* is the number of shared classes. The batch size was set to 36. I used the SGD optimizer, and the initial learning rate and weight decay were set to 0.01 and 0.0005, respectively.

Table 1 shows a comparison of the number of parameters and GFLOPs between the baseline [26] and the proposed method. Since the proposed method in the training phase requires contrastive projection features, additional parameters and GFLOPs of 9.4 and 5.3 M are required. However, for inference, the proposed method uses the same network as the baseline that consists of one feature extractor *G* and one classifier *C*. Notably, the parameters and computational costs for inference do not increase.

### 4.2. Evaluation and Data Augmentation

The goal of the experiments was to compare the proposed results with DANCE [26] across subcases of UDA, i.e., CDA, PDA, and ODA, under synthetic source domain and real target domain classification tasks. Following the evaluation metrics in a previously published study [26], I calculated the accuracy over all target samples in CDA and PDA. In ODA, I used the average per class, including “unknown”. For example, VisDa-2017 ODA reported an average of over seven classes, i.e., six shared and one unknown class. I ran the experiment three times and reported the average results.

I denote the accuracies reported in [26] as DANCE in the tables in this paper. In addition, I present two additional results from [26] as DANCE-R and DANCE-A for a fair comparison. DANCE-R represents the reproduced result of [26] based on their codes (https://github.com/VisionLearningGroup/DANCE. Accessed time: 12 July 2021.) with the same random seeds. DANCE-A represents another trained version of DANCE by the same augmentation μ as that used for the proposed method. This validates my hypothesis that the data augmentation of synthetic source data is not sufficient to cover the real target domain. I set μ to random flip, Gaussian blur, color jitter, and grayscale. I also set $\mu^{\prime }$ to random flip and a scale transform for adjusting the input size to feature extraction network *G* because contrastive learning needs different views generated augmentations μ and $\mu^{\prime }$ based on the same source data.

### 4.3. Datasets and Results

I used VisDA-2017 [36], MNIST [37] and SVHN [38] datasets. VisDA-2017, MNIST, and SNHN were used to analyze the performance of the proposed method in synthetic-to-real environments.

#### 4.3.1. VisDA-2017 Dataset

**Figure 3:**
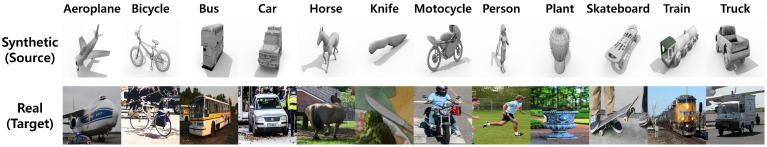
Examples of VisDa-2017. The first row shows synthetic images for the source domain, and the second row shows the real-photographed images. There are substantial variations between the two domains.

VisDA-2017 [36] is a large-scale dataset. It contains 12 category images of various sizes in two domains. One of these contains synthetic 2D renderings of 3D objects with 152,397 images, and the other contains photographs of real-world objects with 55,388 images. I resized the images to 256 × 256 for the feature extraction input. Figure 3 depicts examples of the VisDA-2017 dataset. The first and second rows in Figure 3 depict the source and target domain images, respectively. This dataset exhibits a significant domain shift. I followed [26] to construct closed-set, partial, and open-set domain adaptation tasks to validate the proposed method in large-scale synthetic-to-real domain adaptation. The values in parentheses correspond to the number of shared classes, source private classes, and target private classes, respectively. For instance, (6/6/0) indicates partial domain adaptation, and $|L_{s}\cap L_{t}|$, $|L_{s}-L_{t}|$, and $|L_{t}-L_{s}|$ are 6, 6, and 0, respectively.

As expected, the results in DANCE-* in Table 2 were unfavorable compared to the proposed version in all cases. In particular, the highest improvement was observed in the open-set case. The contrastive loss provides more help since the open-set has many unknown classes. I also checked the effect of hyperparameter $\lambda_{1}$. As shown in Table 2, the proposed method is insensitive to $\lambda_{1}$. In both cases, $\lambda_{1}=0.1$ and $\lambda_{1}=0.03$ were better than the baseline methods. Table 2 also shows a comparison of accuracy between the proposed method and existing other methods. The baseline DANCE-A achieved significantly better accuracy than traditional domain adaptation methods. Nevertheless, the proposed method improved the accuracy of a baseline on VisDa-2017 dataset. Especially for the open-set domain adaptation, learning in the target domain is important; therefore, the proposed method achieved an accuracy improvement of 4.1% over baseline. The proposed method demonstrates almost the same performance as a baseline without significant performance degradation and outperforms other methods.

#### 4.3.2. Digit Datasets: MNIST → SVHN

For another synthetic-to-real domain adaptation scenario, I used two-digit datasets: MNIST [37] and SVHN [38].

**Figure 4:**
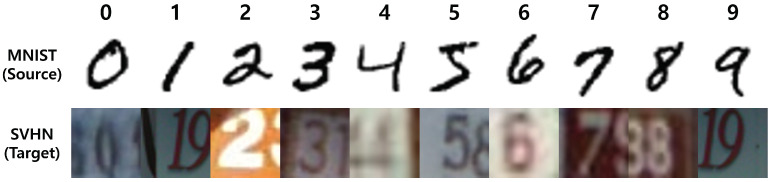
Examples of MNIST and SVHN. The first row shows the MNIST dataset for the source domain. The figures appear synthetic compared to the second-row images in the SVHN dataset acquired from Google Street View. The variations between the two domains are extensive.

MNIST consists of 28 × 28 sized gray images containing handwritten numbers ranging from zero to nine. The standard training and test splits included 60,000 and 10,000 images, respectively. I resized it to 32 × 32 for the experiments. Examples of the images are shown in the first row of Figure 4.

SVHN, the Street View House Numbers dataset, consists of 32 × 32 sized natural scene images of numbers acquired from Google Street View. The training and test splits contain 73,257 and 26,032 images, respectively. The images in the SVHN dataset are aligned at the center of the desired number, but the surroundings are cluttered with visual artifacts and distractors. Examples of the images are depicted in Figure 4.

Images in the MNIST dataset are not synthetic but appear synthetic because they contain only black-and-white image files, as depicted in Figure 4. By contrast, the SVHN images in Figure 4 are obtained from the vision cameras. According to their appearances, I set the training split of MNIST as the source domain and the test split of SVHN as the target domain. I did not apply the random crop, flip, and translation augmentations because the SVHN dataset images also included a second number around the centered number as well as a centered ground-truth number, as depicted in the second row of Figure 4.

For the experiments, I constructed closed-set, partial, and open-set domains as follows: Closed-set domain adaptation: $L_{s}\cap L_{t}=L_{s}=L_{t}=\left\{0,1,2,3,4,5,6,7,8,9\right\}$; Partial domain adaptation: $L_{s}\cap L_{t}=\left\{0,1,2,3,4\right\}$ and $L_{s}-L_{t}=\left\{5,6,7,8,9\right\}$; Open-set domain adaptation: $L_{s}\cap L_{t}=\left\{0,1,2,3,4\right\}$ and $L_{t}-L_{s}=\left\{5,6,7,8,9\right\}$. The results presented in Table 3 are consistent with those of the experiment discussed in Section 4.3.1. DANCE-A shows significantly better results than DANCE-R. Therefore, data augmentation can be used to handle domain gaps. However, the proposed method outperforms DANCE-R and DANCE-A, regardless of the values of hyperparameter $\lambda_{1}$, i.e., for both cases $\lambda_{1}=0.1$ and $\lambda_{1}=0.03$. Even though the baseline DANCE-A uses the same data augmentation as that of the proposed method, the results are, on average, approximately five percent lower than those of the proposed method.

#### 4.3.3. Analysis of the Target Domain Contrastive Loss Function

The results of both experiments in Section 4.3.1 and Section 4.3.2 validated that (1) augmentation of synthetic source domain data does not make sufficient generalization for the target domain even when used in universal domain adaptation algorithms, (2) the added contrastive loss helps generalize the feature space to cover the target domain, and (3) feature network *G* can be shared in target contrastive learning for efficiency.

**Figure 5:**
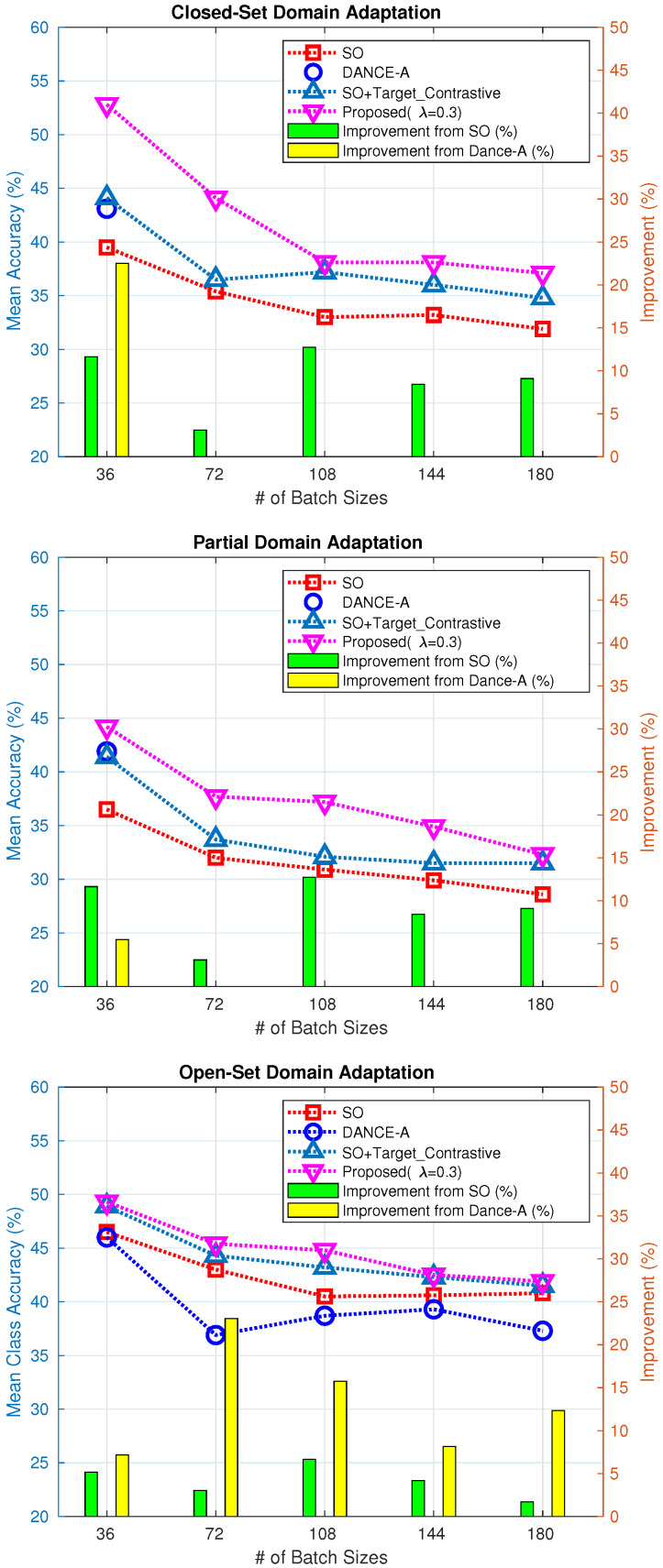
The effect of batch sizes on MNIST to SVHN datasets. ‘SO’, ‘DANCE-A’, and Proposed (λ=0.3) are the same those in Table 3. ‘SO+Target_Contrastive’ means is the addition of target contrastive learning loss to ’SO’. The accuracy improvement rates are represented by green and yellow bars. In the case of optimization unstable, it remains blank.

Figure 5 depicts the effect of batch sizes on MNIST to SVHN datasets, where ‘SO’, ‘DANCE-A’, and Proposed (λ=0.3) are the same those in Table 3. In all comparison methods, regardless of the baseline and proposed method, the accuracy decreased as the batch size increased in closed-set, partial, and open-set domain adaptations. For the domain adaptations, the larger the batch, the more significant the impact of the target domain in the feature space. This makes it difficult for supervised classification features to be transferred from the source domain to the target domain.

I also analyzed the effect of the target domain contrastive learning loss function on domain adaptation. To do this, I added a target domain contrastive learning loss to ‘SO’ that applied unsupervised classification loss function only to the source domain. This is marked ‘SO+Target_Contrastive’. In Figure 5, the cyan triangles consistently demonstrate better accuracy than the red squares, regardless of the batch size. The accuracy improvement rates are represented by green bars. This means that the additional target domain contrastive loss helps the model adapt to the target domain. Thus, the proposed method, which adds a contrastive loss to the baseline DANCE-A, achieved the best result by efficiently learning the target domain. The accuracy improvement rates relative to the baselines are represented by yellow bars. As the batch size increased, the baseline did not optimize the model parameters on close-set and partial domain adaptations; therefore, it remains blank.

## 5. Conclusions

Universal domain adaptation (UDA) is an important research topic for the efficient use of trained models in various image sensors. I found that the baseline method does not have a direct training loss for the target domain to improve the discriminative power. I hypothesized that if the source and target domains are too different, the feature extraction network does not obtain discriminative feature representations for the target domains. To overcome the limitations of the synthetic data, the information about the target domain data should be fully utilized to learn feature representations. To do this, I used contrastive learning [27] in the target domain. The experimental results validated that the proposed method significantly contributed to improving the UDA task. In addition, the target domain feature extraction network was shared with the source domain classification task, avoiding unnecessary computation increases. The proposed method can be easily expanded to help efficient model training on various problems, such as imaging sensors of self-driving cars.

## Figures and Tables

**Table 1:** Comparisons of the number of baseline [26] parameters and GFLOPs during training. For inference, the proposed method uses the same network as the baseline.

Training	Method	Feature Extraction (*G*)	Classification (*C*)	Projection (*M*)	Total
Params	Baseline	23.5 M	0.01 M	-	23.5 M
	Proposed	23.5 M	0.01 M	8.4 M	31.9 M
GFLOPs	Baseline	4.1 × 2	0.0004 × 2	-	8.2
	Proposed	4.1 × 3	0.0004 × 2	0.6 × 2	13.5

**Table 2:** Comparisons of baselines on the VisDA-2017 dataset.

Method (Split)	Closed-Set (12/0/0)	Partial (6/6/0)	Open-Set (6/0/6)	Avg.
SO [26]	46.3	46.3	43.3	45.3
DANN [17]	69.1	38.7	48.2	52.0
ETN [20]	64.1	59.8	51.7	58.5
STA [39]	48.1	48.2	51.7	49.3
UAN [25]	66.4	39.7	50.0	52.0
DANCE [26]	70.2	73.7	65.3	69.7
DANCE-R	71.3	76.6	64.2	70.7
DANCE-A	73.0	80.1	63.8	72.3
Proposed ($\lambda_{1}$ = 0.1)	73.7	82.6	66.7	74.3
Proposed ($\lambda_{1}$ = 0.03)	**74.0**	**83.0**	**67.9**	**75.0**

The source only (SO), DANCE, SO, DANN, ETN, STA, and UAN are the values reported in [26]. The other values are my experimental results.

**Table 3:** Comparison of baselines on MNIST → SVHN.

Method (Split)	Closed Set (10/0/0)	Partial (5/5/0)	Open Set (5/0/5)	Avg.
SO-R	22.7	25.2	30.2	26.0
SO-A	39.5	36.5	46.5	40.8
DANCE-R	25.6	29.1	28.8	27.8
DANCE-A	43.1	41.9	46.0	43.7
Proposed ($\lambda_{1}$ = 0.1)	**53.0**	42.2	48.3	47.8
Proposed ($\lambda_{1}$ = 0.03)	52.8	**44.2**	**49.3**	**48.8**

SO-R and SO-A correspond to the values by a model trained only with source domain samples. DANCE-R and DANCE-A correspond to baselines set [26]. All values represent my experimental results.

## Data Availability

The dataset information. VisDa-2017: https://github.com/VisionLearningGroup/taskcv-2017-public/tree/master/classification; MNIST: http://yann.lecun.com/exdb/mnist/; SVHN: http://ufldl.stanford.edu/housenumbers/.
